# Itinerant Dentistry

**Published:** 1872-05

**Authors:** Joseph H. Scales


### ARTICLE III.

### *Itinerant Dentistry.*

BY JOSEPH H. SCALES, D. D. S.

Being one of a large class of our profession, in which none of our Associations or individual members of prominence seem to take special interest, I am constrained by a sense of duty to myself and fellow laborers, to offer some reflections on this subject. Disclaiming any intention to be facetious or caustic, honesty compels me to say that the proceedings of many of our Associations, and much of the reading offered by our literati, is *wordy*, in fact "a diarrhœa of words and constipation of ideas." They read more like the erratic emanations of an enthusiastic visionary, than sensible, practicable men. I am free to admit that some of the theories proposed and taught are applicable to *located* dentists, but to us they are nonsense, vagaries; not theories.

For instance, supposing the theory to be correct, (which I doubt), how can we afford to treat an alveolar abscess twenty miles from home, when not one in a hundred would submit to it, and not one-tenth of that number be willing to compensate us for it? How often do we hear the remark that "I paid Dr—— $—— to fill my tooth, and when he was gone paid Dr— $— to extract it," when the filling of the tooth was an honest effort to save it, when the nerve was almost or quite exposed.

I can but admire the uncommon common sense of the country people who fail to appreciate, and are not willing to pay for gold crowns built on such foundations as *old roots*, however healthy they may be. In the language of one of the most distinguished members of the profession, "While they show skilful manipulation and manual dexterity, they are nothing more than *walking advertisements*."

The itinerant practitioner is almost daily reminded of the ignorance and consequent want of appreciation in regard to our profession, even among the best educated classes of society. How often do we find it the case, that parents, can converse intelligently upon almost any subject, who do not know how many deciduous teeth their offspring ought to have, or which ones will be replaced. I have on several occasions met persons of more than mediocre intelligence, with well developed bicuspids, who would contend to the last that they had never shed them.

This shows a fearful want of knowledge on the part of parents and others, entrusted with the young during the eruption of the teeth.

Our profession ought to be more prophylactic, and if our "Fathers and mighty men," instead of dissipating their strength in a trial of wind, would, good Samaritan-like, combine and issue a plain, simple, and comprehensible work for "the million," devoid of technicalities, in regard to the management and treatment of the teeth in infancy, during childhood, and even to manhood, I am satisfied great benefit would accrue both to the profession and community at large. When this is done, and not till then, will that parasitic growth on our professional body—empiricism—be extirpated. It lives on ignorance and its concomitant credulity. Remove the cause and the distemper is cured.

I would not be understood as attempting to "throw cold water on," or placing any impediment in the way of scientific experiment, for I am aware that as a branch of the healing art, we have not yet attained our growth. Excel sior has been, and ever will be our motto. I don't believe the *Ultima Thule* ever will be reached, and can only be approached by the advance of experimenting.

It is the duty of every individual member of the profession to contribute his mite for its advancement. Collectively we must dignify dentistry, so as to secure legislative enactments for our protection in every State; secure it upon a legitimate footing, and then like the medical profession, we can "read out" those who vend their *patent* nostrums for filthy lucre.

I believe there are honest, conscientious men in the profession, who to attain the best results in given cases, adapt themselves to circumstances, and although it may not be considered orthodox, sometimes separate with files and chisels, extract sound roots, and even fill with amalgam. I am firmly convinced that in a great many cases, where the operator has not the time or opportunity, and the patient has not the means to pay for it, this is far better than not having it done at all. "Orthodoxy is *my* doxy, and heterodoxy is *your* doxy."

---

